# BCH‐GEE Approach to Examine the Association Between Time‐Varying Food Environment Classes and Multi‐Level Health Outcomes

**DOI:** 10.1002/sim.70550

**Published:** 2026-05-14

**Authors:** Kelsey A. L. Alexovitz, Brisa N. Sánchez, Emma V. Sanchez‐Vaznaugh

**Affiliations:** ^1^ Department of Epidemiology and Biostatistics Dornsife School of Public Health, Drexel University Philadelphia Pennsylvania USA; ^2^ Department of Public Health College of Health and Social Sciences, San Francisco State University San Francisco California USA

**Keywords:** distal outcomes, generalized estimating equations, latent transition analysis

## Abstract

While the creation of latent classes and how they transition over time is useful in describing heterogeneity in a population, examining their relation to other external variables, such as distal outcomes, is often of main interest. There are multiple methods of determining the association between latent classes and distal outcomes. Using modal class assignment as a predictor of the distal outcome is the simplest approach, but these associations suffer from bias due to classification error when assigning latent classes. Other common methods, including the biased‐adjusted 3‐step maximum likelihood or BCH approaches, remove the bias but struggle computationally when estimating associations using complex data structures, such as a larger number of time points or multilevel distal outcomes. We propose a biased‐adjusted 3‐step BCH‐GEE approach that can be used for a multilevel repeated cross‐sectional design. In this approach, latent transition analysis is used to model time‐varying latent classes, followed by ascertainment of classification errors, and weighted GEE to estimate the association between the latent classes and the multilevel distal outcomes. Simulation studies show that the proposed method corrects the bias of the effect estimates that otherwise occur due to the classification error when assigning latent classes. We use this method to examine the association between time‐varying school neighborhood unhealthy food environment classes in California and measures of students' body mass index in 5th grade over an 8 year time‐frame. Results show that children attending schools in neighborhood environments with a higher density of unhealthy food outlets have higher body mass index.

**Abbreviations**
BCH‐GEE: Block Croon Hagenaars—Generalized Estimating Equations
FE: food environment
GEE: generalized estimating equations
LCA: latent class analysis
LTA: latent transition analysis
ML: maximum likelihood
ZIP: zero‐inflated Poisson

## 1 Introduction

**FIGURE 1:**
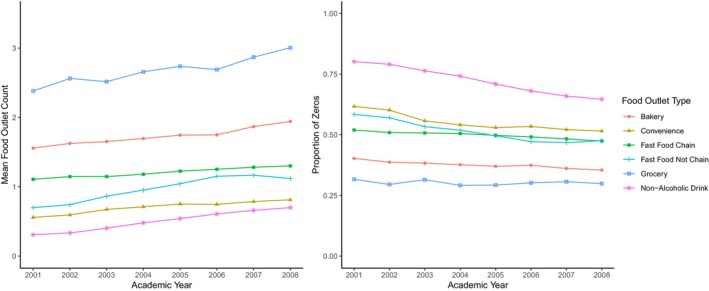
Descriptive statistics of the food outlet counts within 0.5 Miles of 1230 Urban California public schools with 5th Graders. The left panel shows the average count; the right panel shows the proportion of schools with zero outlets within the buffer.

The food environment (FE) is a complex construct involving multiple types of observed features (e.g., availability of different types of food outlets), making it difficult to assess its effects on health. In the project that motivates this paper, we seek to estimate the association between the (possibly time‐varying) food environment near schools and children's body mass index (BMI). Even when we focus solely on unhealthy food outlet availability, there are a variety of different approaches to measuring exposure to the FE, including approaches to addressing challenges given the distribution of the number of available outlets, and characterizing the change in FE. For example, Figure 1 demonstrates the change in the average availability in the number of 6 food outlet types within 0.5 miles of Urban California schools from 2001 to 2018 (left panel), as well as the excess of zero counts within this half‐mile buffer (right panel). We see that over time, the counts increase for all of the food outlet types, and the portion of zero counts is highly dependent on the food outlet type (ranging from 30% to 78%), but overall is also variable over time.

Using a modeling procedure such as latent class analysis (LCA), which creates food environment classes or groupings based on the multiple FE features, enables describing FE exposure in a way that is more readily understood and potentially more actionable. For example, estimating the association between FE classes in a school's neighborhood environment and child obesity would allow policy makers to target areas where interventions would make the most substantial impact (e.g., in areas that are classified as having the worst food environment). Similarly, latent transition analysis (LTA), an extension of LCA for longitudinal data, in addition to identifying latent classes based on multiple features, also allows for modeling the change in FE classes over time [1]. As Figure 1 shows, the availability of these 6 food outlets is variable over time, which provides justification for the use of an LTA rather than an LCA.

Yet, challenges remain regarding how to estimate associations between time‐fixed or time‐varying latent classes and outcomes, particularly when the data structure is complex. Our motivating data represent a complex data structure, in which not only multiple features of the food environment near schools are collected at various time points, but also, at each time point, the BMI of children attending the schools is available for multiple children within the schools.

There are multiple methods to estimate associations between latent classes and distal outcomes (outcomes that depend on latent exposure classes) [2]. Two intuitive, historical methods (that are no longer recommended) are the classify‐analyze approach and the pseudo‐class draw approach. In both of these, a LCA model is used to assign exposure categories for each subject based on the estimated posterior probabilities that each subject belongs to a given latent class; this is followed by a regression of the outcome on the assigned exposure class. Even though they are straightforward to implement, the classify‐analyze approach treats the class assignments as observed and thus fails to account for misclassification (i.e., the assigned class may not be the true latent class). Both of these methods have been shown to result in biased effect estimates [2]. Alternatively, models that simultaneously include the exposure features and the outcomes within a single model can be used to avoid bias related to misclassification error, but these approaches use outcome variables to define the exposure classes, which has been described as problematic in some instances [3].

In more recent years, bias‐adjusted 3‐step methods are able to account and correct for misspecification error, in addition to ensuring that the exposure class definitions do not use outcome data. The two main types of bias‐adjusted methods are the maximum likelihood (ML) method, originated by Vermunt [3], and the BCH method, proposed by Bolck, Croon, and Hagenaars [4] for whom it is named. The BCH method was extended by Vermunt [3] to include explanatory variables other than categorical. Briefly, in a typical scenario of latent exposure classes, the bias‐adjusted 3‐step ML and bias‐adjusted 3‐step BCH methods both follow the same Step 1 and Step 2 calculations, but differ in the way they deal with the class misspecification in Step 3. The steps are: (1) Estimate LCA models with only the latent class and observed exposure features/variables, and choose the number of latent classes using established model selection criteria. (2) Using results from Step 1, determine the assigned latent classes for each subject using the posterior class probabilities for each class for each subject, and estimate the classification error. (3) Estimate the associations between the latent exposure classes and the outcome by fitting a model that now includes the outcome variables (and covariates), along with the assigned latent class and classification errors from Step 2. The specific details for Step 3 depend on whether an ML approach or a BCH approach is used. The ML method includes modeling latent classes, but replaces the observed exposure features with the assigned latent class and sets the conditional response probabilities in the latent exposure model equal to the misclassification error. The BCH method is a weighted pseudo‐likelihood approach that estimates a model for the outcome, using the assigned classes and weights based on the inverse of the misclassification probabilities [2, 5]. In cases of continuous outcomes, BCH is recommended over ML as it is robust to normality assumptions. While both are useful, in cases of complex model structures (e.g., multiple latent class variables), the ML method is more generalizable, while the BCH method can become unwieldy and difficult to use [5, 6]. However, as the model structure becomes increasingly complex (e.g., multilevel analyses), the ML method may also start to suffer from computational difficulties.

These methods have been successfully extended to increasingly complex scenarios, such as LTA models with covariates [7], and multi‐level LCA with covariates [8]. However, limited work is available that models exposures using LTA's and distal outcomes that occur at each time point. In addition, to our knowledge, there is currently no work that takes into account LTA's with a multi‐level repeated cross‐sectional design. Currently, researchers analyzing this type of data must either ignore classification error, introducing substantial bias, or simplify their models by excluding critical features like multi‐level structure or multiple outcomes.

We develop a biased‐adjusted 3‐step BCH‐GEE approach for a repeated cross‐sectional design where a time‐varying latent class exposure variable is available at each repeated cross‐section, along with multilevel outcome data measured at each time point. This method takes into account the classification error when assigning latent classes and thus corrects for the downward bias of the effect estimates that occur, as well as the correlation among outcomes nested within the same time point and/or group. Because the classical bias‐adjusted 3‐step BCH method requires that each subject have a row of data for each possible latent class, when the number of subjects, the number of latent classes, or the number of time points becomes too large (or any combination of the 3), traditional software may struggle to estimate the outcome model. By using a GEE approach in the third step, more complex outcome models can be estimated. Using the zero‐inflated Poisson LTA in our bias‐adjusted 3‐step BCH‐GEE approach, we examine the association between time‐varying school neighborhood food environment classes in California and measures of students' body weight among repeated cross sections of 5th graders.

## 2 Methods

### 2.1 Data

The proposed methods are motivated by a study of the influence of the availability of unhealthy food outlets near schools and body mass index (BMI) among children attending public schools in California (CA). Annual data on public schools in California were obtained from the California Department of Education (CDE), including location, enrollment, and characteristics of the student population [9]. In addition, the characteristics of the census tract where the school was located, such as median household income and percent of adults with 16 or more years of education, were collected from the Census in 2000 and 2010 with the other years linearly interpolated [10, 11]. School locations were classified into urbanization categories based on proprietary Nielsen PRIZM data [12]. Our analyses consisted of 1230 Urban schools that included grade 5.

Child‐level data, including body mass index and demographic data for 823 170 5th graders from the years 2001–2008, were obtained from the CDE's Fitnessgram test, which is an annual physical fitness test [13]. We also retained other child‐level variables, specifically: Race/ethnicity (White, Latino, Black, Asian, and Filipino), fitness status (categories based on not meeting the fitness standard (unfit), meeting the standard (fit), or exceeding it (fit above standard)), age in years, and gender.

Food outlet locations were obtained from the National Establishment Time Series (NETS) database, which provides longitudinal information on businesses across the United States [14]. Food outlet locations for 2001–2008 were obtained for six “unhealthy” food outlet types: (1) fast food chains, (2) fast food non‐chains, (3) convenience stores, (4) small grocers, (5) non‐alcoholic drinking places (including coffee, smoothie, and juice shops) and (6) bakeries (including ice cream, nut and candy shops) based on primary Standard Industry Classification (SIC) codes and chain names [15]. These “unhealthy” food outlet types were selected based on prior research that suggests that these often inexpensive establishments serve high‐calorie and sugary food/drinks, making them especially relevant to students [15, 16, 17]. For each outlet type, the count of outlets within street network buffers of 0.5 miles around California schools was calculated. Differing from Euclidean buffers, which calculate a straight line distance and disregard street connectivity near the location, network buffers calculate these distances based on realistic possible travel paths, such as roads or sidewalks. Prior research regarding buffer areas in the built environment has shown that Euclidean buffers underestimate exposure effects, compared to network buffers [18]. Figure 1 shows descriptive statistics in the form of mean counts and the proportion of zero counts of these “unhealthy” food outlets across all 8 years. We see that the mean counts are variable over time, with an increasing trend, and that the proportion of zeros over time is also variable. While the proportion of zeros differs for each of the 6 “unhealthy” food outlet types, all types exhibit at least 30% zero inflation, with non‐alcoholic drink outlets having a zero‐inflation of up to 78%.

### 2.2 Statistical Methods

**FIGURE 2:**
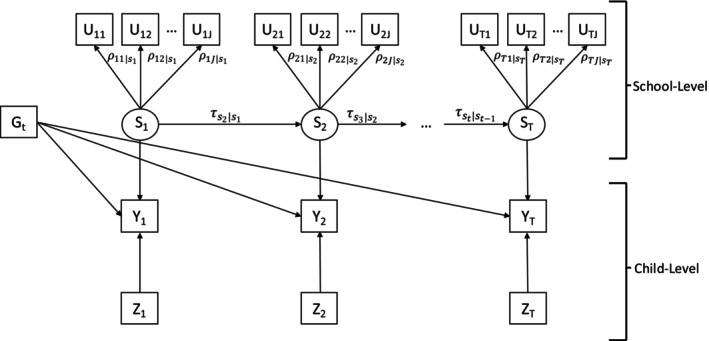
Path diagram for a latent transition analysis model with multiple distal outcomes. Boxes represent observed variables, while circles represent unobserved or latent variables. For the definition of notation, see the text.

Figure 2 shows a path diagram for the overall model that we wish to estimate. Suppressing the index i=1,…,N for schools, for each school, let $U_{t}=(U_{t1},\ldots ,U_{tJ})$ represent a vector of observed counts for food outlets j=1,…,J within 1/2 mile of California public schools at time t, t=1,…T time points. Let $S_{t}$ denote the latent class variable at time t and $s_{t}=1,2,...,L$ be the latent status at time t, where L is the total number of latent classes. The number of latent classes L is taken to be the same at all time points, and although the latent class of the school could change over time, the meaning of the classes is assumed to be the same (i.e., measurement invariant). The school‐level covariates at time t are represented by $G_{t}$. For each school at each time point, let $Y_{t}$ be a vector of distal outcomes. In our motivating example, $Y_{t}$ represents the BMI values of the children who attend the school in the given year t. The vectors $Y_{1}$, … $Y_{T}$, represent T repeated cross sections of children's BMI; that is the BMI of an individual child is not observed longitudinally, but the BMI of children in the same school (i.e., cross‐sections of children in 5th grade) are observed from year to year. Lastly, $Z_{t}$ are the child‐level covariates at time t.

Formally, the model is written as follows, though the subscript for schools, i, is suppressed. First, the latent exposures are modeled at each time point using LCA:

(1) $$Pr[U_{t}=u_{t}]=\overset{L}{\underset{s_{t}=1}{\sum }}P[S_{t}=s_{t}]P[U_{t}=u_{t}|S_{t}=s_{t}]$$

(2) $$=\overset{L}{\underset{s_{t}=1}{\sum }}\pi_{s_{t}}\overset{J}{\underset{j=1}{\prod }}\rho_{t,j|s_{t}}$$

where $\pi_{s_{t}}$ is the probability that latent class $S_{t}$ is $s_{t}$. Assuming conditional independence of the outlet counts given the latent class, $\rho_{t,j|s_{t}}$ represents the conditional response probability for $U_{tj}$ given the latent class. Under the assumption of measurement invariance, these conditional response probabilities are assumed constant over time ($\rho_{t,j|s_{t}}=\rho_{j|s_{t}}$ for all t). Given the zero‐inflated nature of the food outlet counts, we model the conditional response probability, $\rho_{t,j|s_{t}}$, using a zero‐inflated Poisson distribution:

(3) $$\rho_{t,j|s_{t}}=\left\{\begin{array}{ll} p_{js_{t}}+(1-p_{js_{t}})exp(-\lambda_{js_{t}}), & \text{for}\;u=0\\ (1-p_{js_{t}})exp[(u\;ln(\lambda_{js_{t}})-\lambda_{js_{t}})/1-ln(u!)], & \text{for}\;u\geq 1 \end{array}\right.$$

where $p_{js_{t}}$ is the probability of extra zeros and $\lambda_{js_{t}}$ is the expected Poisson count.

Next, we model the marginal association between the latent class at each time point and the outcomes observed at that time point using a GEE approach. We use cell‐means coding to estimate the mean BMI for children attending schools with food environment class status $s_{t}=1,2,...,L$ at time t:

(4) $$Y_{t}=\beta_{1}I(s_{t}=1)+\cdots +\beta_{L}I(s_{t}=L)+G_{t}\gamma_{g}+Z_{t}\gamma_{z}+\epsilon_{t}$$

The associations between food environment classes and the outcome (i.e., differences in BMI associated with different food environment classes) are obtained by subtracting regression coefficients (e.g., $\beta_{L}-\beta_{1}$). The working correlations structure to complete the GEE specification is discussed below.

### 2.3 Bias‐Adjusted 3‐Step BCH‐GEE Method

2.3.1

**FIGURE 3:**
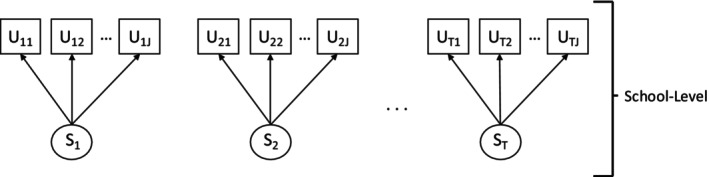
Path diagram for step 1 of the bias‐adjusted 3‐step BCH‐GEE method.

*Overview*. In order to estimate the association between time‐varying latent classes and distal outcomes measured concurrently with the latent classes, we propose a biased‐adjusted 3‐step BCH‐GEE approach. This is a modification of the biased‐adjusted 3‐step BCH approach described by Bakk (2021), among others [2, 5, 19]. While Steps 1 and 2 remain the same as the traditional BCH approach, a GEE formulation is used in Step 3 to estimate the association between the distal outcomes and the assigned latent classes. The steps are as follows:

*Step 1*. Use maximum likelihood to jointly estimate LCA models for each time point, using observed outlet counts ($U_{t}$). By jointly estimating the LCA models, we ensure the classes have the same meaning over all the time points (i.e., measurement invariance). A path diagram for this step is shown in Figure 3, and the mathematical expressions for each LCA are seen in Equations (1) to (3). To determine the appropriate number of classes, models are estimated with a varying number of classes, and the following model fit statistics are compared, in addition to class interpretation: BIC, adjusted BIC, AIC, entropy, and proportion in the smallest class. These are the standard measures reported for studies using LCA. Simulations have shown BIC to perform the best compared to other information criteria, so we use this statistic, along with model interpretation, as the main form of model selection [20]. While the number of classes should not be picked purely based on entropy, entropy (or class separation) should be large enough to ensure optimal model performance (generally around 0.80 or above is considered high entropy) [3].

**FIGURE 4:**
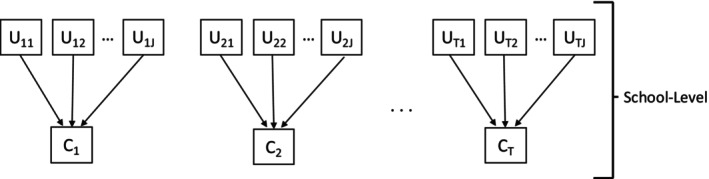
Path diagram for Step 2 of the bias‐adjusted 3‐step BCH‐GEE method.

*Step 2*. Determine every subject's latent class using modal assignment and calculate the classification errors and BCH weights. Specifically, let $C_{t}$ be the assigned latent class membership at time t (as shown in Figure 4), where $C_{t}$ is assigned based on the posterior class probability:

(5) $$P(S_{t}=s_{t}|U_{t}=u_{t})=\frac{P(S_{t}=s_{t})P(U_{t}=u_{t},S_{t}=s_{t})}{P(U_{t}=u_{t})}.$$

While different approaches can be used to make latent class assignments (i.e., “estimate” the latent class), we use modal assignment [19]. That is, we assign each school, at each time point, to the class that has the largest posterior class probability. At this step, we also calculate the conditional probability of the assigned latent class $C_{t}$, given the true latent class $S_{t}$, at a given time point. This probability is often referred to in latent class literature as the classification error, which is what we will also refer to it as. However, it should be noted that if the assigned latent class is equal to the true latent class ($r_{t}=s_{t}$ in Equation 6 below), then the probability is actually the probability of correct classification. In all other scenarios ($r_{t}\neq s_{t}$), it is the classification error. This is given by:

(6) $$q_{r_{t}|s_{t}}=P(C_{t}=r_{t}|S_{t}=s_{t})=\frac{\sum_{i=1}^{n}\sum_{t=1}^{T}P(S_{t}=s_{t}|U_{t}=u_{t})c_{tr}}{N\cdot T\cdot P(S_{t}=s_{t})}$$

where $c_{tr}=P(C_{t}=r_{t}|U_{t}=u_{t})=1$ if the posterior class probability is the largest, and 0 otherwise.

To calculate the BCH weights at each time point, we assemble an L by L matrix of the classification error probabilities, $q_{r_{t}|s_{t}}$:

(7) $$D_{t}=\left[\begin{array}{llll} q_{r_{t}=1|s_{t}=1} & q_{r_{t}=1|s_{t}=2} & \ldots & q_{r_{t}=1|s_{t}=L}\\ q_{r_{t}=2|s_{t}=1} & q_{r_{t}=2|s_{t}=2} & \ldots & q_{r_{t}=2|s_{t}=L}\\ \vdots & \vdots & \ddots & \vdots \\ q_{r_{t}=L|s_{t}=1} & q_{r_{t}=3|s_{t}=2} & \ldots & q_{r_{t}=L|s_{t}=L} \end{array}\right]$$

When misclassification is low, the diagonal classification error probabilities should be close to 1 (i.e., correct classification is in the diagonal entries, per above). The matrix of the BCH weights at each time point, $D_{t}^{*}$, is then given by the inverse of $D_{t}$:

(8) $$D_{t}^{*}={\left(D_{t}\right)}^{-1}=\left[\begin{array}{llll} \omega_{t11} & \omega_{t12} & \ldots & \omega_{t1L}\\ \omega_{t21} & \omega_{t22} & \ldots & \omega_{t2L}\\ \vdots & \vdots & \ddots & \vdots \\ \omega_{t31} & \omega_{t32} & \ldots & \omega_{t33} \end{array}\right]$$

where $\omega_{tr_{t}s_{t}}$ is the BCH weight at time t for a subject assigned to class $r_{t}$ given that their true class is $s_{t}$. The subjects' BCH weights at each time point are comprised of the row of weights that corresponds to their assigned latent class (based on their model class assignment). For example, if at time t a school is assigned to class 1, the weights to be used in Step 3 correspond to the first row of the $D_{t}^{*}$ matrix.

**FIGURE 5:**
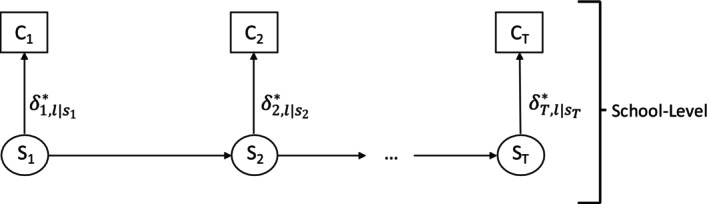
Path diagram for step 3a of the bias‐adjusted 3‐step BCH‐GEE method.

*Step 3a*. Fit an LTA to estimate the probability that a school will change latent classes. As shown in the path diagram in Figure 5, the LTA models the transitions of the true classes $S_{t}$. However, instead of the observed variables ($U_{t}$) being indicators of latent classes, the assigned classes $C_{t}$ are, which not only saves computation time when there are many time points but also enforces measurement invariance assumed in Step 1 and is consistent with the latent class assignments in Step 3b, below. Since $C_{t}$ is a nominal variable, we use a multinomial logistic model, with the response probabilities, $\delta_{t,l|s_{t}}^{*}$, fixed (not estimated) at values based on the classification errors from Step 2. The mathematical expression for this LTA, suppressing a subscript that denotes the subject (in this case, school), is:

(9) $$Pr[C=c]=\overset{T}{\underset{t=1}{\sum }}\overset{L}{\underset{s_{t}=1}{\sum }}\pi_{s_{1}}\left(\tau_{s_{2}|s_{1}}\ldots \tau_{s_{T}|s_{T-1}}\right)\left(\overset{T}{\underset{t=1}{\prod }}\overset{L}{\underset{l=1}{\prod }}\delta_{t,l|s_{t}}^{*}\right)$$

where $\pi_{s_{1}}$ is the initial class probability and $\tau_{s_{t}|s_{t-1}}$ are the transition probabilities. The last component is $\delta_{t,l|s_{t}}^{*}$, which is the conditional response probability, in the form of:

(10) $$logit\left(\delta_{t,l|s_{t}}^{*}\right)\equiv log\left(\frac{q_{r_{t}|s_{t}}}{q_{r_{t}=L|s_{t}}}\right)$$

where class L is the reference/baseline class. Rather than estimating the LTA using only the latent classes assigned $C_{t}$ in Step 2, this approach incorporates a possible misclassification in the latent classes assigned $C_{t}$ from Step 2.

**FIGURE 6:**
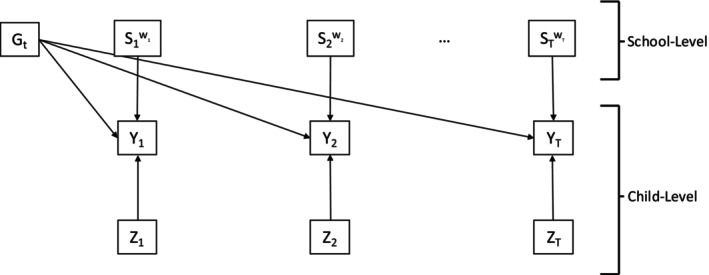
Path diagram for step 3b of the bias‐adjusted 3‐step BCH‐GEE method.

*Step 3b*: Fit a weighted GEE to estimate the marginal association between the latent classes and the outcomes at each time point, using the BCH weights calculated in Step 2. Figure 6 shows the path diagram for this model, where distal outcomes $Y_{t}$ are now included, along with school‐level $G_{t}$ and child‐level $Z_{t}$ predictors of the distal outcome. In the diagram, we use the symbol $S_{t}^{w_{t}}$ to denote that the latent classes $S_{t}$ are replaced by BCH weighted classes for each school based on the model class assignment calculated in Step 2, as described explicitly below. It should be emphasized that $S_{t}^{w_{t}}$, and $G_{t}$ are school level variables, while $Y_{t}$ and $Z_{t}$ are child level variables. This is the only step where child‐level data is being incorporated.

Before describing in more detail the implementation of this step, we first review a naive 3‐step approach (not adjusted for possible misclassification of the modal class assignment). In this case, a GEE for normal outcomes could be used to obtain parameter estimates by solving the estimating equations [21]:

(11) $$𝒮(\beta )=\overset{N_{s}}{\underset{i=1}{\sum }}{X_{i}}^{⊤}{V_{i}}^{-1}(Y_{i}-X_{i}\beta )=0$$

where data vectors and matrices are constructed as follows. The outcome vector for each school is $Y_{i}={\left[\begin{array}{llll} Y_{i1}^{⊤} & Y_{i2}^{⊤} & \ldots & Y_{iT}^{⊤} \end{array}\right]}^{⊤}$ where $Y_{it}$ is a $n_{it}\times 1$ matrix of children's BMI for each school i at time point t, and $n_{it}$ is the number of children at school i for each time point t. The covariate matrix for each school is $X_{i}={\left[\begin{array}{llll} {X_{i1}}^{⊤} & {X_{i2}}^{⊤} & \ldots & {X_{\mathrm{iT}}}^{⊤} \end{array}\right]}^{⊤}$ where $X_{\mathrm{it}}$ is a $n_{it}\times p$ matrix, where p is the total number of school level and child level covariates, including indicator variables for the modal class assignments for each school at each time point.

Lastly, $V_{i}=A_{i}^{1/2}R_{i}(\alpha )A_{i}^{1/2}$ is a $n_{i}\times n_{i}$ covariance matrix formed by the diagonal matrix containing marginal variances, $A_{i}$, and a working correlation matrix $R_{i}(\alpha )$. Depending on which correlation structure is assumed, $V_{i}$ can take different forms. In the Normal case with constant marginal variance ϕ, $A_{i}$ is a diagonal matrix with ϕ on the diagonal. Further, if working independence is assumed (in our case, independence of children within a time point and between time points within a school), the covariance matrix $V_{i}=\phi I_{n_{i}}$. Generally, a non‐independence working correlation structure is assumed since measurements (e.g., BMI of children) within units (e.g., schools) will exhibit correlation. Often, an exchangeable correlation matrix is assumed. In our case, we define a working correlation matrix, $R_{i}(\alpha )$, that is similar to an exchangeable correlation matrix, except that it allows for stronger correlation of BMI values for children measured at the same time point. Said correlation matrix has dimension $n_{i}\times n_{i}$ and can be written as $R_{i}(\alpha )=I_{n_{i}}+\alpha_{w}R_{i,within}+\alpha_{b}R_{i,between}$, where the matrices $R_{i,within}$ and $R_{i,between}$ have values of 0 or 1, with 1 indicating that a particular pair of observations potentially has non‐zero correlation. The first term, $\alpha_{w}R_{i,within}$, models the correlation of observations within school and time point, where the entries in $R_{i,within}$ are 1 only if the children are observed within the same school and time point. The second term models the between‐time point correlation within schools, with the entries of $R_{i,between}$ being 1 only if the pair of observations corresponds to children in the same school, but at different time points. The α values are allowed to be different because observations within a time point may be more highly correlated than observations between time points. Hence, the entries of $R_{i}(\alpha )$ can be formally expressed as:

(12) $$Corr(Y_{itk},Y_{it^{\prime }k^{\prime }})=\left\{\begin{array}{ll} 1, & \text{if}\;t=t^{\prime }\;\text{and}\;k=k^{\prime }\\ \alpha_{w}, & \text{if}\;t=t^{\prime }\;\text{and}\;k\neq k^{\prime }\\ \alpha_{b}, & \text{if}\;t\neq t^{\prime } \end{array}\right.$$

where $k=1,...,n_{it}$ is the child‐level index.

The BCH method for non‐nested data creates a replicate of the outcome data for each potential latent class, and fits the model using all replicates combined, but weighted by the weights created in Step 2 [3, 19]. Hence, for the proposed BCH‐GEE method, we restructure the data by creating L copies of the data, one for each possible class assignment at each time point for each child, $k=1,...,n_{it}$:

(13) $${\tilde{Y}}_{it}={\left[\begin{array}{l} {\tilde{Y}}_{it1}\\ \vdots \\ {\tilde{Y}}_{itn_{it}} \end{array}\right]}_{\left(Ln_{it}\times 1\right)}\;\text{where}\;{\tilde{Y}}_{itk}={\left[\begin{array}{l} Y_{itk}\\ \vdots \\ Y_{itk} \end{array}\right]}_{\left(L\times 1\right)}$$

and

(14) $${\tilde{X}}_{it}={\left[\begin{array}{ll} I_{L} & {\tilde{X}}_{it1}\\ \vdots & \\ I_{L} & {\tilde{X}}_{itn_{it}} \end{array}\right]}_{\left(Ln_{it}\times (L+p)\right)}\;\text{where}\;{\tilde{X}}_{itk}={\left[\begin{array}{l} X_{itk}\\ \vdots \\ X_{itk} \end{array}\right]}_{\left(L\times p\right)}.$$

The inclusion of $I_{L}$ in the covariate matrix ${\tilde{X}}_{\mathrm{it}}$ allows us to note that each of the L rows for child k refers to a different possible class assignment. The covariate matrix ${\tilde{X}}_{\mathrm{itk}}$ includes L copies of the school‐ and child‐level covariate row vector ($X_{itk}$) but does not contain the indicator variables for the modal class assignments. In addition, BCH weights are included as:

(15) $${\tilde{W}}_{i}=diag{\left({\tilde{W}}_{i1},\ldots ,{\tilde{W}}_{iT}\right)}_{\left(Ln_{i}\times Ln_{i}\right)}$$

where

(16) $$\begin{array}{ll} {\tilde{W}}_{it} & =diag{\left({\tilde{W}}_{it1},\ldots ,{\tilde{W}}_{itn_{it}}\right)}_{\left(Ln_{it}\times Ln_{it}\right)}\;\text{and}\\ \;{\tilde{W}}_{itk} & =diag{\left(\omega_{tr_{t}1},\ldots ,\omega_{tr_{t}L}\right)}_{\left(L\times L\right)} \end{array}$$

We obtain parameter estimates by solving weighted estimating equations [21]:

(17) $$𝒮(\beta )=\overset{N_{s}}{\underset{i=1}{\sum }}{\tilde{X}}_{i}^{⊤}{\tilde{V}}_{i}^{-1}{\tilde{W}}_{i}({\tilde{Y}}_{i}-{\tilde{X}}_{i}\beta )=0$$

In the Normal case with constant marginal variance ϕ, if working independence is assumed, the covariance matrix is ${\tilde{V}}_{i}=\phi I_{\left(Ln_{i}\right)}$. If a non‐independence working correlation structure is assumed, ${\tilde{V}}_{i}$ is written as:

(18) $${\tilde{V}}_{i}={Ã}_{i}^{1/2}{\tilde{R}}_{i}(\alpha ){Ã}_{i}^{1/2}$$

where ${Ã}_{i}=\phi I_{\left(n_{it}L\right)}$ and ${\tilde{R}}_{i}(\alpha )$ is a $\left(n_{it}L)\times (n_{it}L\right)$ modified exchangeable correlation matrix that allows for different magnitude of correlation within and between time points. The correlation matrix is expressed similarly to Equation 12, however, the following correlations have to be set to zero: Correlations within a child between classes, and among all children measured within the same time point but between classes [22]. Consider the l and $l^{\prime }$ copies of outcome values $Y_{itk}$, $Y_{itkl}$ and $Y_{it^{\prime }k^{\prime }l^{\prime }}$. The entries of the correlation matrix for the expanded dataset are:

(19) $$Corr(Y_{itkl},Y_{it^{\prime }k^{\prime }l^{\prime }})=\left\{\begin{array}{ll} 1, & \text{if}\;t=t^{\prime }\;\text{and}\;k=k^{\prime }\;\text{and}\;l=l^{\prime }\\ \alpha_{w}, & \text{if}\;t=t^{\prime }\;\text{and}\;k\neq k^{\prime }\;\text{and}\;l=l^{\prime }\\ 0, & \text{if}\;t=t^{\prime }\;\text{and}\;k=k^{\prime }\;\text{and}\;l\neq l^{\prime }\\ 0, & \text{if}\;t=t^{\prime }\;\text{and}\;k\neq k^{\prime }\;\text{and}\;l\neq l^{\prime }\\ \alpha_{b}, & \text{if}\;t\neq t^{\prime } \end{array}\right.$$

where $k=1,...,n_{it}$ is the child‐level index.

(20) $$\begin{array}{ll} & {\tilde{R}}_{i,within}=diag[{\tilde{R}}_{i,within,1},\ldots ,{\tilde{R}}_{i,within,T}]\text{, where}\\ & {\tilde{R}}_{i,within,t}=-I_{Ln_{it}}+{\left[\begin{array}{ccc} I_{L} & \ldots & I_{L}\\ \vdots & \ddots & \vdots \\ I_{L} & \ldots & I_{L} \end{array}\right]}_{\left(Ln_{it}\times Ln_{it}\right)} \end{array}$$

(21) $${\tilde{R}}_{i,between}=1_{Ln_{i}}1_{Ln_{i}}^{T}-diag(1_{Ln_{i1}}1_{Ln_{i1}}^{T},\ldots ,1_{Ln_{iT}}1_{Ln_{iT}}^{T})$$

The estimating algorithm for the BCH‐GEE, with re‐configured data as in the above equations, is as follows:

1. Let ℓ=0. Start by using a weighted GEE assuming working independence to obtain initial estimates $\hat{\beta^{\left(0\right)}}$:
(22) $$\hat{\beta^{\left(0\right)}}={\left(\overset{N_{s}}{\underset{i=1}{\sum }}{\tilde{X}}_{i}^{⊤}{\tilde{W}}_{i}{\tilde{X}}_{i}\right)}^{-1}\left(\overset{N_{s}}{\underset{i=1}{\sum }}{\tilde{X}}_{i}^{⊤}{\tilde{W}}_{i}{\tilde{Y}}_{i}\right)$$
2. Let ℓ=ℓ+1. For each school i in $i=1,...,N_{s}$ calculate:
(23) $$\text{Residuals:}\;e_{i}={\tilde{Y}}_{i}-{\tilde{X}}_{i}\hat{\beta^{\left(\ell \right)}}$$
(24) $$\text{Scale parameter:}\;\phi =\frac{\sum_{i=1}^{N}{e_{i}}^{⊤}{\tilde{W}}_{i}e_{i}}{N_{total}-p}$$
(25) $$\text{Working Covariance:}\;{\tilde{V}}_{i}={Ã}_{i}^{1/2}{\tilde{R}}_{i}(\alpha ){{Ã}_{i}}^{1/2}$$
  where $N_{total}$ is the total number of children across all schools and time points, p is the number of regression parameters (including one for each class), ${Ã}_{i}=\phi I$ and ${\tilde{R}}_{i}(\alpha )$ is the correlation matrix. If a working independence correlation structure is assumed, $R_{i}(\alpha )=I$. If an exchangeable correlation structure is assumed, α parameters for between‐school and within time point correlations must be estimated:
(26) $$\begin{array}{ll} & \text{Within school and time point correlation:}\\ & \;\alpha_{w}=\frac{\sum_{i=1}^{N}diag(e_{i}){\tilde{R}}_{i,within}{\tilde{W}}_{i}diag(e_{i})}{\phi (N_{w}/L-p)} \end{array}$$
(27) $$\begin{array}{ll} & \text{Between school correlation:}\\ & \;\alpha_{b}=\frac{\sum_{i=1}^{N}diag(e_{i}){\tilde{R}}_{i,between}{\tilde{W}}_{i}diag(e_{i})}{\phi (N_{b}/L-p)} \end{array}$$
  where $N_{w}$ are the number of non‐zero entries in ${\tilde{R}}_{i,within}$ and $N_{b}$ are the number of non‐zero entries in ${\tilde{R}}_{i,between}$.
3. Estimate $\hat{\beta^{\left(\ell \right)}}$ using working covariance matrix ${\tilde{V}}_{i}$ estimated in Step 2:
(28) $$\hat{\beta^{\left(u\right)}}={\left(\overset{N_{s}}{\underset{i=1}{\sum }}{\tilde{X}}_{i}^{⊤}{\tilde{V}}_{i}^{-1}{\tilde{W}}_{i}{\tilde{X}}_{i}\right)}^{-1}\left(\overset{N_{s}}{\underset{i=1}{\sum }}{\tilde{X}}_{i}^{⊤}{\tilde{V}}_{i}^{-1}{\tilde{W}}_{i}{\tilde{Y}}_{i}\right)$$
4. Iterate between Steps 2 and 3 until convergence is achieved:
(29) $$max\left|\hat{\beta^{\left(u\right)}}-\hat{\beta^{\left(u-1\right)}}\right|<\epsilon$$
  where ϵ is a suitably small number. Finally, calculate the sandwich variance estimator:
(30) $$\begin{array}{ll} Cov\left(\hat{\beta^{\left(u\right)}}\right) & ={\left(\overset{N}{\underset{i=1}{\sum }}{\tilde{X}}_{i}^{⊤}{\tilde{V}}_{i}^{-1}{\tilde{W}}_{i}{\tilde{X}}_{i}\right)}^{-1}\\ & \;\times \left(\overset{N}{\underset{i=1}{\sum }}{𝒮}_{i}\left(\hat{\beta^{\left(u\right)}}\right){𝒮}_{i}{\left(\hat{\beta^{\left(u\right)}}\right)}^{⊤}\right)\\ & \;\times {\left(\overset{N}{\underset{i=1}{\sum }}{\tilde{X}}_{i}^{⊤}{\tilde{V}}_{i}^{-1}{\tilde{W}}_{i}{\tilde{X}}_{i}\right)}^{-⊤} \end{array}$$
(31) $$\text{where}\;{𝒮}_{i}\left(\hat{\beta^{\left(u\right)}}\right)={\tilde{X}}_{i}^{⊤}{\tilde{V}}_{i}^{-1}{\tilde{W}}_{i}\left({\tilde{Y}}_{i}-{\tilde{X}}_{i}\hat{\beta^{\left(u\right)}}\right).$$

## 3 Simulation Study

We conducted a simulation study to evaluate the ability of the proposed bias‐adjusted 3‐step BCH‐GEE method to estimate associations between a time‐varying latent class exposure and distal outcomes measured concurrently with the latent class indicators (i.e., a repeated cross‐sectional design). In particular, we want to compare this approach with a standard 3‐step GEE (Equation 11), which does not take into consideration the classification errors. We vary three components to evaluate how they affect the performance of these methods: (1) the working correlation structure of the GEE, (2) separation between classes (quantified by entropy), and (3) effect size (difference in the outcome means between latent class exposures). We also investigate whether the probability of classes transitioning has an impact on the performance of these methods. We compare these methods' performance based on three measures regarding the outcome model parameters (β's): % bias, MSE, and variance (both the sandwich estimator variance and the variance of the parameter estimates). R code for implementing each method and conducting the simulation study is provided at https://github.com/kelsey‐alexo/2026‐StatMed‐BCH‐GEE‐Approach.

### 3.1 Data Generation

First, we generate 500 datasets for each of the four scenarios formed by the combination of entropy and effect size. Each dataset has 1230 schools (like in the California dataset). To generate each dataset, we assume that there are three latent classes, measured through three observed variables (i.e., three food outlet types). Values for these observed variables were generated from a zero‐inflated Poisson distribution with parameters set to ensure our two entropy values; ZIP distribution parameters are shown in Appendix A, Table A1. Entropy is a measure of class separation and is determined based on the parameter values of the conditional response probability model. When entropy is high, classes are well separated and are less likely to be misclassified. When entropy is low, classes are more likely to be misclassified, which may affect parameter estimates. We consider two scenarios: A “high” entropy of 0.79, and a “low” entropy of 0.52.

Initial class probabilities were set to be approximately equal among the 3 classes. The transition probabilities were set so that schools had a high probability (>92%) of staying in their initial class, as we have in the CA data. In a second simulation, we investigate what happens when transition probabilities are set so that schools have a higher probability of transitioning between classes, which may occur in different types of data (63%–74%). Outcomes were generated by first simulating an average school outcome at each of the 4 time points, using:

(32) $$\bar{Y_{it}}=\beta_{1}Class1_{it}+\beta_{2}Class2_{it}+\beta_{3}Class3_{it}+b_{i}+b_{it}$$

where Class1–Class3 are indicators for the generated true latent class. Random effects $b_{i}∼N(0,\sigma_{b}^{2})$ and $b_{it}∼N(0,\sigma_{w}^{2})$ were used to induce an exchangeable correlation structure with both within and between time point correlations, as defined previously. Then, child‐level outcomes for child k were generated as:

(33) $$Y_{itk}=\bar{Y_{it}}+\beta_{4}z_{itk}+\epsilon_{itk}$$

where $\epsilon_{itk}∼N(0,\sigma^{2})$, and $z_{itk}$ is a binary child level predictor. The number of children within each school at each time point varies between 20–80. In this model, we varied the effect size to determine if the proposed method has difficulty correctly estimating parameters when the effect size is small. Because of two different sources of variance (random effects and person‐level error), we define effect size as the difference in the outcome means between latent class exposures divided by the marginal standard deviation, e.g., $(\beta_{i}-\beta_{j})/\sqrt{\sigma^{2}+\sigma_{b}^{2}+\sigma_{w}^{2}}$. Using effect size enables us to have a sense of the magnitude of the associations that is less tied to the coefficient and variance values (i.e., a “standardized coefficient”). For the smaller effect size (0.05), we used $\beta_{1}=22$, $\beta_{2}=21.87$, and $\beta_{3}=21.74$, yielding pairwise differences of 0.13. For the larger effect size (0.25), we used $\beta_{1}=22$, $\beta_{2}=21.35$, and $\beta_{3}=20.7$, yielding pairwise differences of 0.65. We set $\beta_{4}=0.20$ for all scenarios. In order for the simulations to run in a reasonable time frame, $\sigma^{2}$ was chosen to be smaller than the actual $\sigma^{2}$ exhibited in our California data (i.e., less noise in the simulated data leads to faster convergence). However, $\sigma_{b}^{2}$ and $\sigma_{w}^{2}$ were chosen to accurately reflect the between time point ($\alpha_{b}$) and within school and time point ($\alpha_{w}$) correlations that are seen in the California data. These correlations are calculated as the proportion of each of the given random effect variances to the total variance.

Finally, since misclassification of latent classes can affect both regression coefficient estimates and their variances, potentially leading to biased inference, we conducted an additional simulation study under the null effect size (ES = 0) to assess type I error. Specifically, we set $\beta_{1}=\beta_{2}=\beta_{3}=0$ to obtain an effect size of 0, and used a Wald test using the sandwich variance estimates with a significance level of 0.05.

### 3.2 Estimation

We estimated model parameters as described in section 2, for each dataset. The newly proposed bias‐adjusted 3‐step BCH‐GEE and standard 3‐step GEE methods were estimated assuming two different covariance structures: Working independence and exchangeable correlation.

### 3.3 Results

Table 1 shows the performance measures when working independence and exchangeable correlation structures are used. As we are mainly interested in the differences in the outcome associated with the latent classes, we focus on examining the difference in class regression coefficients, as well as covariance parameter estimates. Performance measures for the individual parameters ($\beta_{1}\;\text{to}\;\beta_{4}$) are available in Appendix Table A2.

**TABLE 1:** Performance measures for working independence and exchangeable correlation structure (MSE and variances are multiplied by 1000 for readability).

				BCH‐GEE				GEE			
Entropy^a^	E.S.^b^	Param	True Value	% Bias^c^	MSE^d^	Var ^e^	Var ^f^	% Bias^c^	MSE^d^	Var ^e^	Var ^f^
*Independence*											
0.52	0.05	$\beta_{1}-\beta_{2}$	0.13	−6.9	4.9	4.8	4.5	−28.3	2.5	1.2	1.0
		$\beta_{2}-\beta_{3}$	0.13	−2.8	3.9	3.9	4.3	−13.8	1.4	1.1	1.1
		ϕ	6.76	0.0	0.7	—	—	0.1	0.7	—	—
	0.25	$\beta_{1}-\beta_{2}$	0.65	−5.1	14.1	13.1	5.3	−27.2	34.3	3.0	1.2
		$\beta_{2}-\beta_{3}$	0.65	−4.4	12.0	11.2	5.1	−14.6	12.1	3.1	1.2
		ϕ	6.76	0.3	3.5	—	—	1.5	10.6	—	—
0.79	0.05	$\beta_{1}-\beta_{2}$	0.13	−4.5	2.3	2.3	2.3	−16.7	1.9	1.4	1.2
		$\beta_{2}-\beta_{3}$	0.13	0.1	2.0	2.0	2.3	1.7	1.5	1.4	1.3
		ϕ	6.76	0.0	0.7	—	—	0.0	0.7	—	—
	0.25	$\beta_{1}-\beta_{2}$	0.65	−2.2	4.4	4.2	2.4	−14.9	10.8	1.5	1.2
		$\beta_{2}-\beta_{3}$	0.65	−1.6	4.8	4.7	2.4	0.3	1.5	1.5	1.3
		ϕ	6.76	0.1	1.7	—	—	0.5	2.0	—	—
*Exchangeable*											
0.52	0.05	$\beta_{1}-\beta_{2}$	0.13	−7.1	4.6	4.5	4.3	−46.2	4.5	0.9	0.8
		$\beta_{2}-\beta_{3}$	0.13	−2.3	3.8	3.8	4.1	−34.5	3.0	1.0	0.9
		$\alpha_{w}$	0.08	0.1	0.0	—	—	−18.6	0.2	—	—
		$\alpha_{b}$	0.05	0.1	0.0	—	—	−37.8	0.4	—	—
		ϕ	6.76	0.0	0.7	—	—	0.1	0.7	—	—
	0.25	$\beta_{1}-\beta_{2}$	0.65	−5.2	13.9	12.8	5.0	−46.9	95.4	2.4	1.0
		$\beta_{2}-\beta_{3}$	0.65	−4.2	12.0	11.3	4.8	−35.3	56.1	3.3	1.1
		$\alpha_{w}$	0.08	4.1	0.1	—	—	−3.6	0.0	—	—
		$\alpha_{b}$	0.05	5.2	0.0	—	—	−27.	0.2	—	—
		ϕ	6.76	0.3	3.5	—	—	1.6	13.3	—	—
0.79	0.05	$\beta_{1}-\beta_{2}$	0.13	−4.3	2.2	2.2	2.2	−29.6	2.7	1.2	1.0
		$\beta_{2}-\beta_{3}$	0.13	0.0	1.9	2.0	2.2	−6.0	1.4	1.4	1.2
		$\alpha_{w}$	0.08	0.1	0.0	—	—	−19.0	0.2	—	—
		$\alpha_{b}$	0.05	0.0	0.0	—	—	−38.2	0.4	—	—
		ϕ	6.76	0.0	0.7	—	—	0.0	0.7	—	—
	0.25	$\beta_{1}-\beta_{2}$	0.65	−2.2	4.3	4.1	2.3	−28.6	35.9	1.4	1.2
		$\beta_{2}-\beta_{3}$	0.65	−1.6	4.8	4.7	2.3	−5.9	3.1	1.6	1.4
		$\alpha_{w}$	0.08	1.6	0.0	—	—	−14.2	0.1	—	—
		$\alpha_{b}$	0.05	2.0	0.0	—	—	−35.6	0.3	—	—
		ϕ	6.76	0.1	1.7	—	—	0.6	2.2	—	—

^a^ Entropy is defined as a measure of class separation.

^b^ Effect size (E.S.) is defined as the difference in the outcome means between latent class exposures $\left(\beta_{i}-\beta_{j}\right)$ divided by the marginal standard deviation.

^c^ % Bias is defined as the mean difference in the observed vs. true values, divided by the true value and multiplied by 100 to make it a percentage.

^d^ Mean Squared Error (MSE) is defined as the mean squared difference between the observed values and the true values, multiplied by 1000 for readability.

^e^ ${\mathrm{Var}}^{e}$ is the variance of the parameter estimates multiplied by 1000 for readability.

^f^ ${\mathrm{Var}}^{f}$ is the variance based on the sandwich estimator multiplied by 1000 for readability.

Focusing first on working independence correlation structure, we note that although BCH‐GEE does not completely eliminate bias, it has considerably less bias than the naive GEE. The naive GEE can have biases up to 28%, while the BCH‐GEE bias is less than 7% for all effect sizes and entropy levels. Thus, BCH‐GEE provides an improvement over available methods. For both the BCH‐GEE and GEE methods, as class separation (entropy) increases, the % bias decreases, especially for the naive GEE. Hence, when entropy is high, the difference between the methods is smaller. Within the same level of entropy, changes in effect size have minimal impact on % bias. However, note that the bias for $\beta_{2}-\beta_{3}$ is consistently smaller than the bias for $\beta_{1}-\beta_{2}$, which is especially evident for the naive GEE in the case of low entropy. This is due to differences in missclassification probabilities (entries) across classes and entropy levels (example $D_{t}$ matrices shown in Appendix Table A3). In the low entropy case, the probability of correctly classifying subjects into Class 1 is around 57%, but 81%–89% for Classes 2 and 3. Hence, there is greater bias for $\beta_{1}-\beta_{2}$. Additionally, in the low entropy case, there is a sizable missclassification between Class 1 and Class 3 of up to 11%–20%, which also contributes to bias since Class 1 is the “highest exposure” class (with larger average outcome) and Class 3 is the “lowest exposure” class. However, this misclassification between classes 1 and 3 is negligible in the high entropy case. Thus, while in the high entropy case we see biases in $\beta_{1}-\beta_{2}$, the bias in $\beta_{2}-\beta_{3}$ is reduced due to less missclassification between Class 1 and 3. Note that the empirical variances of the estimates are similar for $\beta_{1}-\beta_{2}$ and $\beta_{2}”\beta_{3}$ for the same effect size; however, the corresponding MSEs have large differences in magnitudes driven by the differences in bias.

Comparing the sandwich variance estimator (${\mathrm{Var}}^{f}$) to the variance of the parameter estimates (${\mathrm{Var}}^{e}$), we see that the BCH‐GEE method underestimates the parameter uncertainty when the effect size is large (0.25) and more closely estimates it when the effect size is small (0.05). For the GEE method, we see that the sandwich estimator underestimates parameter uncertainty, further exacerbating problems with inference given the large bias. The variances for the GEE method are smaller than the variances for the BCH‐GEE method, as may be expected given that BCH‐GEE accounts for missclassification error.

We see similar patterns in the exchangeable correlation structure as we do in the working independence correlation structure: As entropy increases, % bias decreases, and the BCH‐GEE method has much smaller % biases than the GEE method, particularly when entropy is small. The main difference is that the GEE method performs worse using an exchangeable correlation structure, with % biases up to ∼47%, while for the BCH‐GEE method, it performs similarly well. When comparing the variances of the estimates (${\mathrm{Var}}^{e}$) across the two correlation assumptions, we see that using exchangeable correlations leads to generally slightly smaller variances than working independence, as would be expected.

In Table 1, we set parameters so that the probability of staying in the initial class is high (>92%), like we see in the application data. Table 2 shows the results for a working independence correlation structure when there is a smaller probability of staying in the initial class (∼63%–74%). In this case, we see that for both the BCH‐GEE and GEE methods, % bias is smaller, though the difference is greater for the GEE method.

**TABLE 2:** Performance measures for working independence correlation structure when transition probabilities are greater (MSE and variances are multiplied by 1000 for readability).

				BCH‐GEE				GEE			
Entropy^a^	E.S.^b^	Param	True Value	% Bias^c^	MSE^d^	Var ^e^	Var ^f^	% Bias^c^	MSE^d^	Var ^e^	Var ^f^
0.79	0.05	$\beta_{1}-\beta_{2}$	0.13	−1.7	1.8	1.8	1.7	−14.1	1.5	1.1	0.9
		$\beta_{2}-\beta_{3}$	0.13	−2.6	2.0	2.0	1.7	−0.4	1.4	1.4	1.1
		ϕ	6.76	<0.1	0.7	—	—	<0.1	0.6	—	—
	0.25	$\beta_{1}-\beta_{2}$	0.25	−1.8	3.8	3.7	1.8	−14.4	10.0	1.2	1.0
		$\beta_{2}-\beta_{3}$	0.25	−2.5	5.2	5.0	1.8	0.0	1.4	1.4	1.1
		ϕ	6.76	0.1	1.9	—	—	0.5	2.0	—	—

^a^ Entropy is defined as a measure of class separation.

^b^ Effect size (E.S.) is defined as the difference in the outcome means between latent class exposures $\left(\beta_{i}-\beta_{j}\right)$ divided by the marginal standard deviation.

^c^ % Bias is defined as the mean difference in the observed vs. true values, divided by the true value and multiplied by 100 to make it a percentage.

^d^ Mean Squared Error (MSE) is defined as the mean squared difference between the observed values and the true values, multiplied by 1000 for readability.

^e^ ${\mathrm{Var}}^{e}$ is the variance of the parameter estimates multiplied by 1000 for readability.

^f^ ${\mathrm{Var}}^{f}$ is the variance based on the sandwich estimator multiplied by 1000 for readability.

Table 3 shows the results of the evaluation of Type I error for both entropy levels and correlation structures. Across all scenarios, we found that the BCH‐GEE maintained Type I error at or below the nominal 5% level. In contrast, the GEE approach exhibited slightly inflated Type I error in all the scenarios, with empirical rejection rates between 0.056–0.076.

**TABLE 3:** Performance measures for working independence and exchangeable correlation structure, when Effect Size is 0 (Bias, MSE, and variances are multiplied by 1000 for readability).

			BCH‐GEE					GEE				
							Type I					Type I
Entropy^a^	E.S.^b^	Param	Bias^c^	MSE^d^	Var ^e^	Var ^f^	Error	Bias^c^	MSE^d^	Var ^e^	Var ^f^	Error
*Independence*												
0.52	0	$\beta_{1}-\beta_{2}$	−2.8	4.4	4.4	4.4	0.044	−1.5	1.2	1.2	1.0	0.070
		$\beta_{2}-\beta_{3}$	3.3	3.6	3.6	4.1	0.030	1.9	1.1	1.1	1.1	0.056
0.79	0	$\beta_{1}-\beta_{2}$	−3.1	2.2	2.2	2.3	0.042	−2.5	1.5	1.5	1.2	0.076
		$\beta_{2}-\beta_{3}$	3.2	1.9	1.9	2.2	0.036	2.9	1.5	1.5	1.3	0.070
*Exchangeable*												
0.52	0	$\beta_{1}-\beta_{2}$	−2.8	4.1	4.1	4.2	0.042	−0.1	0.9	0.9	0.8	0.064
		$\beta_{2}-\beta_{3}$	3.7	3.4	3.4	3.9	0.028	−0.1	0.9	0.9	0.9	0.070
0.79	0	$\beta_{1}-\beta_{2}$	−2.8	2.1	2.1	2.2	0.040	−0.9	1.2	1.2	1.0	0.068
		$\beta_{2}-\beta_{3}$	3.0	1.8	1.8	2.2	0.034	0.1	1.4	1.4	1.2	0.076

^a^ Entropy is defined as a measure of class separation.

^b^ Effect size (E.S.) is defined as the difference in the outcome means between latent class exposures $\left(\beta_{i}-\beta_{j}\right)$ divided by the marginal standard deviation.

^c^ Bias is defined as the mean difference in the observed vs. true values.

^d^ Mean Squared Error (MSE) is defined as the mean squared difference between the observed values and the true values, multiplied by 1000 for readability.

^e^ ${\mathrm{Var}}^{e}$ is the variance of the parameter estimates multiplied by 1000 for readability.

^f^ ${\mathrm{Var}}^{f}$is the variance based on the sandwich estimator multiplied by 1000 for readability.

## 4 Examining the Association Between Children's BMI and School Food Environment

In this section, we apply the bias‐adjusted 3‐step BCH‐GEE model with a working independence correlation structure to examine the relationship between the unhealthy food environment surrounding California public schools in urban areas and BMI in 5th graders from 2001 to 2008, as described in Section 2.1. We aim to (1) create classes to categorize the unhealthy food environment surrounding Urban California schools, (2) determine how likely schools are to transition from one food environment class to another, and (3) estimate the effects of these food environment classes on the BMI of 5th graders.

Observed unhealthy food outlet counts for six outlet types within a 0.5‐mile network buffer of 1230 urban California schools that were open between 2001 and 2008 were used in our analysis. School‐level characteristics include the percentage of students who receive free and reduced‐priced meals (% FRPM), median household income of the school's census tract (Median Income (per 10k)), and percent of adults with 16 or more years of education in the school's census tract (% Education). BMI values for 823 170 5th graders along with their categorical child‐level characteristics (sex, fitness status, race/ethnicity, and age) were obtained from the CDE's Fitnessgram test.

**FIGURE 7:**
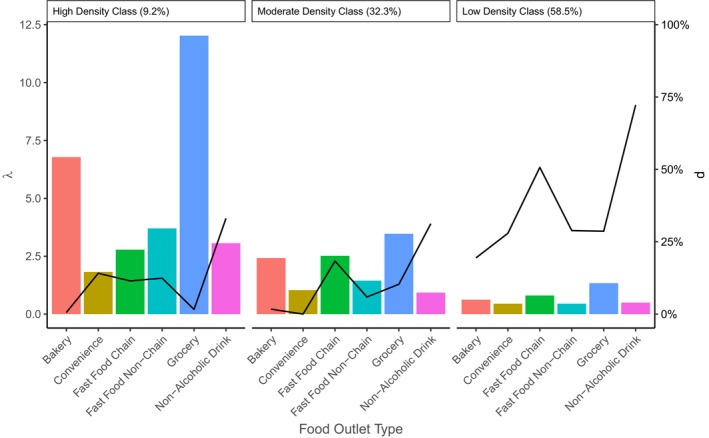
Estimated conditional response probability for Urban 5th grade schools. The height of bars indicates values of λ for each outlet type, while line p indicates the probability of zero inflation. The percentage of schools in each class at the first time point, 2001, is given next to the class number.

A 3‐class model was chosen by comparing model fit statistics for models with 2 to 5 classes. While those with greater than 3 classes had smaller BICs, since the proportion of schools in the 4th and/or 5th classes was <1%. This means that one of the classes is made up of only a handful of schools, which leads to issues calculating BCH weights. All models had high entropy (>80%). The zero‐inflated Poisson conditional response probability parameters for the 3‐class model can be seen in Figure 7. These classes are measured by the unhealthy food outlet counts surrounding schools. Class 1 has the largest mean counts for each unhealthy food outlet and smaller amounts of zero inflation, and is labeled as the “high density unhealthy FE” class. It is also the class with the smallest proportion of schools (9.2%) at the first time point, 2001. Class 2 is labeled as the “moderate density unhealthy FE” class, as the mean counts are between the values for Class 1 and Class 3. It contains 32.3% of schools in 2001, and Class 3 is the “low density unhealthy FE” class. It has the largest amount of zero inflation, with small mean counts, and is made up of a majority of schools (59.6%). Therefore, fewer schools are exposed to environments with the highest obesogenic potential, and a majority are exposed to school food environments with the lowest potential.

Table 4 gives the school characteristics and child characteristics for each class in 2001. Schools in the high‐density unhealthy FE class have more students who receive free and reduced‐price meals (FRPM), and are located in census tracts with lower median household income and a lower proportion of individuals who have received a bachelor's degree, than the lower density unhealthy FE classes. The racial/ethnic makeup of the 5th graders of schools in the high density unhealthy FE class shows that they are comprised of more Latino children and fewer White children than schools in the other classes.

**TABLE 4:** Characteristics of Urban 5th grade schools in each class at 2001 (Baseline).

	High density class	Moderate density class	Low density class
School characteristics, *N* (%)	113 (9.2%)	397 (32.3%)	720 (58.5%)
% FRPM ^a^, median (IQR)	92.9 (81.0–96.6)	76.6 (50.4–91.4)	63.0 (27.3–84.0)
% Education ^b^, median (IQR)	8.0 (3.6–19.2)	14.3 (7.2–26.2)	17.8 (8.9–32.5)
Median Income (per 10k), median (IQR)	2.8 (2.2–3.6)	4.1 (3.2–5.1)	4.9 (3.9–6.4)
**Student Characteristics, N (%)**	14 357 (14.0%)	32 924 (32.1%)	55 224 (53.9%)
Female, *n* (%)	7110 (49.5)	16 312 (49.5)	26 882 (48.7)
Age, *n* (%)			
10	8886 (61.9)	20 184 (61.3)	32 938 (59.6)
11	5246 (36.5)	12 189 (37.0)	21 352 (38.7)
12	225 (1.6)	551 (1.7)	934 (1.7)
Fitness Status, *n* (%)			
Unfit	5933 (41.3)	13 673 (41.5)	22 768 (41.2)
Fit	5938 (41.4)	13 330 (40.5)	21 693 (39.3)
Fit Above Standard	2486 (17.3)	5921 (18.0)	10 763 (19.5)
Race/Ethnicity, *n* (%)			
White	278 (1.9)	4749 (14.4)	14 027 (25.4)
Latino	12 119 (84.4)	21 960 (66.7)	30 955 (56.1)
Black	1031 (7.2)	4.036 (12.3)	5719 (10.4)
Asian	708 (4.9)	1888 (5.7)	3959 (7.2)
Filipino	221 (1.5)	291 (0.9)	564 (1.0)

^a^ % FRPM is the percentage of students who receive free and reduced‐priced meals in the school.

^b^ % Education is the percent of adults with 16 or more years of education in the school's census tract.

The probability of schools transitioning unhealthy FE classes over the full 8‐year time period is shown in Table 5. Over an 8‐year period, schools were relatively unlikely to transition out of their unhealthy FE class, as the probability of transitioning to another class ranges from 0%–17%. The probability that a school's unhealthy FE class worsens over time (transitions from a low density to a moderate density or a moderate density to a high density) is slightly greater than the probability that it will improve. There is a 0% probability that schools transition from a high density unhealthy FE to a low density unhealthy FE. We explored whether allowing transition probabilities varied between time points, as shown in Table A4. The transition probabilities were largely similar, with a maximum range of transition probabilities of ∼5%.

**TABLE 5:** Transition probabilities for Urban schools over 8 years.

		Class in 2008 High density	Moderate density	Low density
**Class in 2001**	**High density**	0.85	0.15	0.00
	**Moderate density**	0.14	0.83	0.03
	**Low density**	0.01	0.17	0.82

Two models were estimated to examine the effects of unhealthy FE class on the BMI of 5th graders: Model 1 adjusts for child‐level covariates, while Model 2 adjusts for both child and school‐level covariates. Since simulation results showed the sandwich variance estimator exhibits some difficulty estimating parameter uncertainty, we also estimated the standard error using bootstrapping. We generated 500 bootstrap replicates by sampling 1230 schools with replacement in Step 2 of the BCH‐GEE and GEE methods. Table 6 shows the parameter estimates and standard errors for both models. There is a positive association between 5th graders' BMI and the density of the school's unhealthy FE class. For both Models 1 and 2 in the BCH‐GEE method, we see that 5th graders' BMI is lower when the children attend schools in an FE class that reflects a lower density of unhealthy food outlets. There is a larger difference in BMI when comparing children attending schools in the high density unhealthy FE class to those in the low density class (0.41 for Model 1), than when comparing those attending in high vs. moderate FE density classes (0.15 for Model 1). When adjusting for school‐level covariates in addition to child‐level covariates, these associations were slightly attenuated. Overall, the GEE method shows a slightly smaller association between 5th graders' BMI and the density of schools' unhealthy FE class compared to the BCH‐GEE approach, as would be expected.

**TABLE 6:** Difference in BMI among children attending schools with a food environment with high vs. moderate or low density of unhealthy food outlets.

	BCH‐GEE			GEE		
Variable	Estimate	SE ^a^	SE ^b^	Estimate	SE ^a^	SE ^b^
*Model 1: Child‐level covariates*						
High ‐ Moderate Density Classes	0.15	0.08	0.08	0.16	0.07	0.07
High ‐ Low Density Classes	0.41	0.07	0.08	0.38	0.07	0.08
*Model 2: Child and school‐level covariates*						
High ‐ Moderate Density Classes	0.13	0.08	0.08	0.12	0.07	0.08
High ‐ Low Density Classes	0.24	0.08	0.09	0.20	0.07	0.08

*Note*: The table shows parameter estimates and standard errors from the BCH‐GEE and conventional GEE methods assuming a working independence correlation structure, adjusted for child‐level covariates only (Model 1), or both child and school‐level covariates (Model 2).

^a^ ${\text{SE}}^{a}$ is the SE based on the sandwich estimator.

^b^ ${\text{SE}}^{b}$ is the bootstrapped SE based on 500 replicates.

## 5 Discussion

In this paper, we present a bias‐adjusted 3‐step BCH‐GEE approach for estimating the marginal association between time‐varying latent class exposures and distal outcomes at multiple time points, within a multi‐level cross‐sectional design. While previous methods are theoretically capable of estimating these associations, they struggle computationally. We showed using simulations that using a weighted GEE with BCH weights in Step 3 of the proposed method removes bias that occurs when using an unweighted GEE approach, especially when there is strong separation between latent classes and when there is a larger probability of transitioning between latent classes over time. We applied this method to examine the association between the unhealthy food environment around urban public schools in California and BMI for 5th graders attending those schools. We found that schools can be classified into 3 unhealthy FE classes: High density, moderate density, and low density, and that as the density of the schools' unhealthy food environment class increases, 5th graders' BMI also increases.

The proposed BCH‐GEE method is an extension of the bias‐adjusted 3‐step BCH and ML methods [3, 4], which were developed to correct for bias that arises due to using the modal class assignment instead of the true classes in the model for the distal outcome (i.e., bias from the misclassification error). While these original methods are theoretically able to be used in very complex scenarios (>3 time points, large sample sizes, multiple distal outcomes), their likelihoods become increasingly complex and computationally intensive to estimate. For example, in the case of the original bias‐adjusted 3‐step BCH method, joint weights over all the time points need to be calculated (i.e., $L^{T}$ weights). For the model presented in section 4 (with 3 classes and 8 time points), this would require 6561 joint BCH weights, while our GEE‐BCH approach only requires 24 (i.e., L·T). Hence, even when it would potentially be ideal to use them, they are not currently feasible. Our proposed BCH‐GEE provides an approach to reduce bias compared to a naive GEE method, which is the only other feasible comparison given the absence of available ML or BCH 3‐step correction approach for multilevel data. Comparing our BCH‐GEE approach to a naive/unweighted GEE approach demonstrates the impact of ignoring classification error. An important direction for future methodological research is the development of a computationally feasible ML‐based 3‐step approach for multi‐level LTA with distal outcomes. Full ML approaches consider missclasification probabilities jointly across time points, which may provide improvements over GEE‐BCH, such as further removal of bias and increased efficiency.

We chose to use a GEE approach, rather than a linear mixed effects (LME) model for the multi‐level data for two reasons. Firstly, in this case, we are interested in estimating the marginal or population‐averaged associations, not subject specific association; thus, a GEE is appropriate. While a random effects model could be used to model these associations instead of a GEE approach, the interpretation of coefficients from both approaches is equivalent since we are using an identity link function with an assumption of symmetrical mean‐zero error distribution. In addition, since we are using this method specifically for a repeated cross‐sectional design, if a mixed model approach were used, only school‐level random effects could be conditioned on for the purpose of modeling the correlation (i.e., no child‐level random effects). This can also be done using GEE, which has a much simpler formulation in this scenario. Secondly, a full mixed model including latent class modeling would be computationally unfeasible, as the likelihood would require integrating the latent classes and each distal outcome over all of the time points. In the case of many time points, the computation becomes exceedingly complex. In addition, an algorithm such as the standard EM or Baum–Welch is needed to maximize the likelihood when random effects are included [7]. Hence, a GEE approach addresses the research question and is also computationally feasible.

The proposed method suffers from some of the same limitations as preceding BCH approaches. For instance, if a class has a very small proportion of subjects for any of the time points, BCH weights may not be able to be calculated, and this method cannot be used. Thus, in practice, the selection of the number of latent classes in the LCA also needs to consider this issue. In the California dataset, we chose the 3‐class model (over 4 or 5 classes) in part for this reason. Additionally, as the sample size, number of classes, and number of time points increase, computational power issues and difficulties with convergence may occur. These issues may also occur in Step 1 of the method, as the conditional response probability model chosen for the observed variables becomes more complex. Nevertheless, our approach enables the estimation of interest compared to prior approaches, where estimation would not be computationally feasible.

In applying this method to California public schools, we found that schools have a small probability of transitioning unhealthy FE classes over time. This informed our GEE approach to modeling the effect of these classes on children's BMI by including an indicator for each of the classes that were measured concurrently with the outcomes. If we had found that schools' unhealthy FE classes were more variable over time, latent classes from the previous time point could potentially be added to the outcome model to determine whether schools' class transitioning to a less or more dense class had an additional effect on children's BMI. However, additional work is needed to implement this idea. For example, at each time point, the BCH weights may need to be modified to account for the uncertainty of the class assignment at time t and at time t−1.

There are different class assignment rules based on the posterior class probability that can be used to create the assigned latent class in Step 2 of our proposed method. We used modal assignment, which assigns each school to the class that has the largest posterior class probability. Another commonly used assignment rule is proportional assignment, which assigns weights that equal the posterior class probability for all possible class patterns for each school and time point. Bakk et al. [19] compared the results for the bias‐adjusted 3‐step BCH and ML methods using modal and proportional class assignment. They found that when entropy is at least moderate (≥0.65), and sample size is large (>1000), both class assignment methods perform similarly well. When entropy is very low, and/or the sample size is small, proportional assignment performs slightly better than modal. We choose to use modal assignment since we have a large sample size. In practice, entropy values of <0.65 are not recommended, and retaining the proportional assignments weights increases the size of our already large dataset needed for estimation. However, the use of proportional assignment should be straightforward to implement within the BCH‐GEE framework and could be explored in future work.

The results of the simulation study offer guidance for applying the proposed approach more generally. In the simulation study, we compared two correlation structures, working independence and exchangeable structures, in Step 3b of the proposed method. We found that the % bias of the coefficient estimates for these two correlation structures was very similar, but the variances were slightly smaller for the exchangeable correlation structure, as would be expected. When deciding which correlation structure to use, computational power and time should be considered. For large complex datasets, the estimation algorithm for Step 3b may take a significant amount of time and computational power to converge if using the multilevel exchangeable correlation structure we proposed. The simulation study also found that the sandwich variance estimator may overestimate or underestimate parameter uncertainty. To address this issue, we used bootstrap standard errors to derive inferences for the California data in Section 4. We bootstrap replicates in Step 2 of our method (i.e., when constructing the BCH weights after fitting the LCA). We choose to sample in Step 2 to ensure that the meaning of each of the latent classes established in Step 1 would remain the same for all replicates. Resampling in Step 1, before creating the latent classes, could result in differing class meanings as well as class label switching between replicates.

In sum, the proposed biased‐adjusted 3‐step BCH‐GEE offers an alternative to current methods of estimating the relationship between time‐varying latent classes and distal outcomes. The approach extends the type of data scenarios in which these methods can be implemented.

## Funding

This work was supported in part by the National Institutes of Health (NIH), specifically the National Institute on Minority Health and Health Disparities (R01MD017687), the National Heart, Lung and Blood Institute (R01HL136718, R01HL131610), and the Eunice Kennedy Shriver National Institute of Child Health and Human Development (R01HD111169).

## Conflicts of Interest

The authors declare no conflicts of interest.

## Data Availability

The data that support the findings of this study are available from the California Department of Education. Restrictions apply to the availability of these data, which were used under license for this study. Data are available from the author(s) with the permission of the California Department of Education.

## Appendix A TABLES

**TABLE A1:** Parameters for the conditional response probability models used to generate three observed variables, for 3 latent classes.

Entropy	Parameters	U	U	U
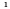
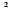
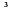
Low—0.52	$\lambda_{1}(p_{1})$	4.6 (0.15)	5.6 (0.10)	5.1 (0.04)
	$\lambda_{2}(p_{2})$	2.8 (0.27)	3.8 (0.13)	3.3 (0.35)
	$\lambda_{3}(p_{3})$	1.0 (0.70)	2.0 (0.40)	1.5 (0.50)
High—0.79	$\lambda_{1}(p_{1})$	10.4 (0.15)	11.4 (0.10)	10.9 (0.04)
	$\lambda_{2}(p_{2})$	5.7 (0.27)	6.7 (0.13)	6.2 (0.35)
	$\lambda_{3}(p_{3})$	1.0 (0.70)	2.0 (0.40)	1.5 (0.50)

*Note*: For each variable, the Poisson parameter λ is followed by the probability of zero inflation p.

**TABLE A2:** Performance measures for working independence and exchangeable correlation structure (MSE and variances are multiplied by 1000 for readability).

				BCH‐GEE				GEE			
Entropy^a^	E.S.^b^	Param	True Value	% Bias^c^	MSE^d^	Var^e^	Var^f^	% Bias^c^	MSE^d^	Var^e^	Var^f^
*Independence*											
0.52	0.05	$\beta_{1}$	22.00	−0.03	1.70	1.65	1.39	−0.12	1.52	0.80	0.63
		$\beta_{2}$	21.87	0.01	2.20	2.20	2.13	0.05	0.99	0.89	0.64
		$\beta_{3}$	21.74	0.02	1.47	1.45	1.38	0.13	1.57	0.80	0.65
		$\beta_{4}$	0.20	0.10	0.10	0.10	0.11	0.09	0.10	0.10	0.11
	0.25	$\beta_{1}$	22.00	−0.14	8.88	7.88	1.58	−0.59	18.65	1.56	0.69
		$\beta_{2}$	21.35	0.01	6.35	6.36	2.54	0.22	6.07	3.94	0.76
		$\beta_{3}$	20.70	0.15	8.83	7.94	1.59	0.68	21.64	1.74	0.72
		$\beta_{4}$	0.20	0.20	0.10	0.10	0.11	0.14	0.10	0.10	0.11
0.79	0.05	$\beta_{1}$	22.00	−0.02	1.09	1.08	1.07	−0.05	1.00	0.86	0.68
		$\beta_{2}$	21.87	0.01	1.13	1.13	1.19	0.05	0.97	0.87	0.69
		$\beta_{3}$	21.74	0.01	1.09	1.08	1.11	0.04	0.95	0.89	0.73
		$\beta_{4}$	0.20	0.04	0.10	0.10	0.11	0.03	0.10	0.10	0.11
	0.25	$\beta_{1}$	22.00	−0.05	2.90	2.77	1.11	−0.24	3.70	0.91	0.70
		$\beta_{2}$	21.35	0.01	1.28	1.27	1.25	0.21	2.88	0.96	0.73
		$\beta_{3}$	20.70	0.06	4.08	3.91	1.14	0.20	2.66	0.92	0.75
		$\beta_{4}$	0.20	0.08	0.10	0.10	0.11	0.05	0.10	0.10	0.11
*Exchangeable*											
0.52	0.05	$\beta_{1}$	22.00	−0.03	1.65	1.60	1.34	−0.23	3.36	0.72	0.58
		$\beta_{2}$	21.87	0.01	2.12	2.12	2.02	0.04	0.80	0.73	0.56
		$\beta_{3}$	21.74	0.02	1.43	1.41	1.34	0.25	3.67	0.81	0.60
		$\beta_{4}$	0.20	0.04	0.10	0.10	0.11	0.04	0.10	0.10	0.10
	0.25	$\beta_{1}$	22.00	−0.14	8.82	7.85	1.51	−1.19	69.86	1.75	0.68
		$\beta_{2}$	21.35	0.01	6.34	6.35	2.39	0.21	4.33	2.39	0.70
		$\beta_{3}$	20.70	0.14	8.88	7.99	1.52	1.32	77.30	2.36	0.73
		$\beta_{4}$	0.20	0.02	0.10	0.10	0.11	0.04	0.10	0.10	0.10
0.79	0.05	$\beta_{1}$	22.00	−0.02	1.04	1.03	1.05	−0.12	1.45	0.77	0.66
		$\beta_{2}$	21.87	0.01	1.11	1.11	1.16	0.06	0.97	0.82	0.64
		$\beta_{3}$	21.74	0.01	1.04	1.04	1.08	0.09	1.33	0.92	0.74
		$\beta_{4}$	0.20	−0.02	0.10	0.10	0.11	−0.01	0.10	0.10	0.10
	0.25	$\beta_{1}$	22.00	−0.05	2.85	2.73	1.08	−0.57	16.36	0.83	0.72
		$\beta_{2}$	21.35	0.01	1.27	1.27	1.21	0.29	4.68	0.95	0.71
		$\beta_{3}$	20.70	0.07	4.05	3.87	1.11	0.48	10.87	1.01	0.80
		$\beta_{4}$	0.20	−0.03	0.10	0.10	0.11	0.00	0.10	0.10	0.10

^a^ Entropy is a measure of class separation.

^b^ Effect size (E.S.) is defined as the difference in the outcome means between latent class exposures ($\beta_{i}-\beta_{j}$) divided by the marginal standard deviation.

^c^ % Bias is defined as the mean difference in the observed vs true values, divided by the true value and multiplied by 100 to make it a percentage.

^d^ Mean Squared Error (MSE) is defined as the mean squared difference between the observed values and the true values, multiplied by 1000 for readability.

^e^ Var is the variance of the parameter estimates multiplied by 1000 for readability.

^f^ Var is the variance based on the sandwich estimator multiplied by 1000 for readability.

**TABLE A3:** Entries in the classification probability matrix $D_{t}$ in a single simulation iteration when effect size is 0.25.

	Assigned class in low entropy scenario			Assigned class in high entropy scenario		
True class	Class 1	Class 2	Class 3	Class 1	Class 2	Class 3
Class 1	0.572	0.225	0.203	0.972	0.028	0.000
Class 2	0.165	0.806	0.029	0.065	0.856	0.079
Class 3	0.108	0.008	0.885	0.001	0.080	0.918

*Note*: The rows show the true latent class and the columns show the class the subject is assigned to.

**TABLE A4:** Range of transition probabilities for Urban schools over all timepoints.

		Class at timepoint *t*		
	Class	Class 1	Class 2	Class 3
**Class at timepoint *t*‐1**	Class 1	0.942–1.000	0.000–0.058	0.000
	Class 2	0.000–0.038	0.957–1.000	0.000–0.018
	Class 3	0.000	0.000–0.041	0.959–1
